# Can Chaotic Analysis of Electroencephalogram Aid the Diagnosis of Encephalopathy?

**DOI:** 10.1155/2018/8192820

**Published:** 2018-05-29

**Authors:** Jisu Elsa Jacob, Ajith Cherian, K. Gopakumar, Thomas Iype, Doris George Yohannan, K. P. Divya

**Affiliations:** ^1^Department of Electronics and Communication Engineering, SCT College of Engineering, Thiruvananthapuram, Kerala, India; ^2^Department of Neurology, SCTIMST, Thiruvananthapuram, Kerala, India; ^3^Department of ECE, TKM College of Engineering, Kollam, Kerala, India; ^4^Department of Neurology, Government Medical College, Thiruvananthapuram, Kerala, India; ^5^Department of Anatomy, Government Medical College, Thiruvananthapuram, Kerala, India

## Abstract

Chaotic analysis is a relatively novel area in the study of physiological signals. Chaotic features of electroencephalogram have been analyzed in various disease states like epilepsy, Alzheimer's disease, sleep disorders, and depression. All these diseases have primary involvement of the brain. Our study examines the chaotic parameters in metabolic encephalopathy, where the brain functions are involved secondary to a metabolic disturbance. Our analysis clearly showed significant lower values for chaotic parameters, correlation dimension, and largest Lyapunov exponent for EEG in patients with metabolic encephalopathy compared to normal EEG. The chaotic features of EEG have been shown in previous studies to be an indicator of the complexity of brain dynamics. The smaller values of chaotic features for encephalopathy suggest that normal complexity of brain function is reduced in encephalopathy. To the best knowledge of the authors, no similar work has been reported on metabolic encephalopathy. This finding may be useful to understand the neurobiological phenomena in encephalopathy. These chaotic features are then utilized as feature sets for Support Vector Machine classifier to identify cases of encephalopathy from normal healthy subjects yielding high values of accuracy. Thus, we infer that chaotic measures are EEG parameters sensitive to functional alterations of the brain, caused by encephalopathy.

## 1. Introduction

The electroencephalogram (EEG) is one of the methods to study the “brain at work” in real time. The EEG is a signal generated from the brain which reflects the sum total of excitatory and inhibitory postsynaptic potentials of large population of cortical neurons. The use of EEG as an adjuvant to clinical diagnosis of neurological diseases has drifted from mere visual observation of the waveforms to complex analysis of the EEG signal using well-defined algorithms. Objective analysis of EEG is being parlayed similar to efforts made in radiology to rule out individual bias. To date in clinical practice, EEG observation is the standard, for all neurological conditions. The use of newer methods in EEG analysis gives objective evidence and shows its potential to be used for comparison in various clinical scenarios. As the EEG signal is in the order of microvolt and often affected by many artefacts like eye and muscle movements, highly efficient signal processing algorithms may be utilized to explore the subtle information embedded in it. One of the emerging techniques in the field of analysis of EEG is to extract the chaotic features in the EEG signal and utilize these indicators for diagnosing various neurological diseases.

A few studies in the past have performed nonlinear analysis of EEG in neurological diseases like epilepsy [1–5], Alzheimer's disease [6–8], depression, and other mental diseases [9, 10]. Research has also been conducted on analysis of nonlinear features of EEG in various stages of sleep [11] and stages of anaesthesia [12, 13]. Many of the above works have reported positive findings. The basic theory behind conducting nonlinear analysis in EEG is that EEG signal is produced fundamentally due to a nonlinear deterministic process between various neurons which are highly dynamic in nature [14]. Nonlinear property is exhibited even at the neuronal level [15]. Since brain activity is the sum total of interactions of millions of neuronal populations, nonlinear analysis is an apt approach for EEG study [16]. Chaotic analysis, which forms a subset of nonlinear analysis, is therefore well suited for studying EEG signals and its nature.

The pioneering works of chaotic analysis of EEG were reported in seizure disorders and epilepsy as early as in 1980s [17–20]. Those works highlighted the lowering of chaotic parameter, correlation dimension (CD), during seizure when compared with that of normal EEG, suggesting that seizures might be due to a pathological “loss of complexity,” which made them describe seizure as a state of “low dimensional chaos.” Brain, being an intricate network of coupled and dynamic interacting subsystems, a higher brain function like cognition is highly dependent on the efficient integration and processing of signals in the network. The complexity of the brain dynamics increases when a subject performs a cognitive task. The brain dynamics during various cognitive tasks were studied extensively using nonlinear analysis. These studies have also attempted to show the relationship between brain dynamics complexity to the type and difficulty of the specific task. Several studies have described an increase in the CD during cognitive tasks [21, 22] which are “higher brain functions.” The tasks used in the studies included arithmetic, visual, verbal, graphemic, and memory retrieval. Another study revealed the correlation of EEG complexity with the difficulty level of the cognitive task [23]. The studies on normal cognition were extended to the application of nonlinear analysis to neurological diseases characterized by disturbed cognition, in particular dementia [24]. Studies on patients with Alzheimer's disease, a common type of dementia, demonstrated a loss of dynamical complexity [6, 25].

While many of the studies mentioned above demonstrate the decreasing trend in chaotic features in various types of neurological diseases and an increasing pattern during a higher brain function, the pattern of chaotic features during encephalopathy remains unexplored. Metabolic encephalopathy can be defined as fluctuating or reversible global change in brain function manifesting with impairment of attention, disturbances in the circadian sleep-wake cycle, deficits in higher level brain functions, and changes in arousal [26]. It is a disease condition when the normal activity of brain is affected either temporarily or permanently due to malfunctioning of some other organs of the body. Various types of encephalopathy are defined based on the primary cause of brain affliction. To the best of our knowledge, similar data and results are sparse in metabolic encephalopathy. This study aims at applying chaotic analysis of EEG in patients with encephalopathy and comparing its features with that of normal EEG and thereby to probe whether chaotic features are good indicators for diagnosing this disease.

## 2. Materials and Methods

### 2.1. Data Acquisition

#### 2.1.1. Study Design

The study design was retrospective case-control study.

#### 2.1.2. Study Setting

EEG data used in this study has been taken from the EEG Lab, Department of Neurology, Government Medical College, Thiruvananthapuram, Kerala, India. Research committee approval and ethics committee clearance of the institution were obtained prior to the conduct of the study.

#### 2.1.3. Study Sample with Inclusion and Exclusion Criteria

*Cases*. EEG data of 30 patients with metabolic encephalopathy were recorded. The patients with metabolic encephalopathy aged more than 18 years were included for the study. Patients with structural pathology, infections of the CNS, and cerebral vascular insult (confirmed by neuroimaging or other investigations) and patients with clinical picture suggestive of metabolic encephalopathy but without obvious metabolic disturbances detected in the necessary biochemical investigations and metabolic encephalopathy occurring in the background of another neurological illness causing cognitive dysfunction or a degenerative condition were excluded from our study. Most of the cases were in a state of delirium during EEG recording. The EEG signals of patients were recorded without any medication.

*Controls*. These selected cases were compared with a control group of 30 normal individuals. Patients who came with single episode of syncope but were clinically normal and having normal brain imaging, where seizures and structural lesions were ruled out, have been enrolled as normal healthy controls.

#### 2.1.4. Selection of Specific Epochs of EEG Recordings for Analysis

Artefact free regions in the EEG were identified by neurologists, specially trained in interpretation of EEG, for our study. These selected areas of each EEG recordings were saved as epochs of a continuous stretch, each of 12-second duration. As the sampling frequency was fixed at 500 Hz during recording, each EEG epoch contained approximately 6000 sampling points. Around 8–12 epochs of EEG were obtained for each EEG recording, in both normal and encephalopathy groups. This range was set as the availability of artefact free “clean EEG” stretches of 12-second duration was variable in different recordings. EEG was recorded using average reference montage in the Nicolet EEG machine using NicVue v.3.0 software. 10–20-electrode system was adopted for the EEG tracing with 21-channel recording. The contacts used were Fp1, Fp2, F3, F4, C3, C4, P3, P4, F7, F8, T3, T4, T5, T6, O1, O2, A1, A2, Fz, Cz, and Pz in addition to the ground electrode, EKG, and EMG electrodes. 314 EEG epochs of normal subjects were compared with 331 epochs of EEG of patients with encephalopathy. Tables 1 and 2 give the details of demographic data of patients and details of the epochs taken for this analysis, respectively.

### 2.2. Chaotic Feature Extraction

The data that was recorded was preprocessed by removing artefacts by passing through band pass filter to extract the area of interest, i.e., 0.5 Hz to 60 Hz. EEG data was then saved as epochs of 12-second duration in text files in ASCII format. These 12 s EEG epochs were processed in MATLAB for extracting chaotic features. All the technical work related to this study and computation of features were done using MATLAB R2014a which is a high level technical computing software and used extensively in signal processing and numerical integration.

Biosignals are nonstationary and dynamic in nature. There is an innate variability in the normal physiological state of human body, which is a dynamic system. In other words, a normal biosignal is inherently “chaotic” in nature. A shift from this, to a more ordered or “less chaotic” system, can indicate a diseased state [27]. So, chaotic analysis of these signals has given better results when compared to time-domain and frequency-domain analysis. This is the basic assumption which underlies the attempts in conducting chaotic analysis in EEG signals.

The EEG signal is represented as a one-dimensional time series vector *x*(*n*) = {*x*_1_, *x*_2_,…, *x*_*N*_}, where *N* is the number of sampling points and the subscripts indicate the time instant of the data point.

This one-dimensional signal has to be modelled in an *m*-dimensional phase space or state space (*Rm*). Takens introduced a method explaining how to reconstruct the model in phase space [28]. This method is called Takens' method of delays. This method is employed till now in the field of nonlinear analysis. The EEG epoch *x*(*n*) is modelled in an *m*-dimensional Euclidean space as

(1) $$\begin{array}{c} X_{m}\left(\;n\;\right)=\left[\;x\left(\;n\;\right),x\left(\;n-\lambda \;\right),\ldots ,x\left(\;n-\left(\;m-1\;\right)\lambda \;\right)\;\right], \end{array}$$

where *λ* is the time delay and *m* is the embedding dimension (vide infra). The time delay and embedding dimension are called embedding parameters. Thus, each vector in *m*-dimensional state space consists of *m* coordinates [29]. In case of dissipative deterministic dynamical systems where energy is lost with time, various trajectories will converge to a subspace of the total state space, which is called attractor, when the system is observed for a long time [29]. The subspace is called attractor because it is supposed to converge (or attract) trajectories from all possible initial conditions. The dynamics corresponding to a strange attractor is called deterministic chaos.

In state-space reconstruction, the major requirement is to select the optimum value for embedding parameters *λ*  and *m*.

#### 2.2.1. Estimation of Time Delay (*λ*)

Time delay can be calculated from autocorrelation method or mutual information method. *λ*  is the time (in samples) of the first zero crossing of autocorrelation function. The mutual information method to find optimum *λ* was proposed by Fraser and Swinney [30, 31].

Mutual information is

(2) $$\begin{array}{c} s=-\overset{n}{\underset{k=0}{\sum }}p_{ij}\left(\;\lambda \;\right)\ln\frac{p_{ij}}{p_{i}p_{j}}. \end{array}$$

As *λ*  increases, *s* decreases and then again increases. Optimum delay is taken as the time delay when mutual information, *s*, reaches its first minimum.

#### 2.2.2. Estimation of Embedding Dimension (*m*)

False Nearest Neighbor (FNN) method is commonly employed for calculating optimum value of embedding dimension. It was suggested by Kennel et al. based on the assumption that if the attractor is constructed successfully in *m*-dimensional space, all points that are close will also be sufficiently close in *m* + 1 dimensional space [32]. A point that does not satisfy is considered as false neighbor. The number of false neighbors is calculated for increasing value of *m*. The value of *m* at which false neighbors become zero or decrease drastically is taken as the optimum value of embedding dimension *m*.

#### 2.2.3. Correlation Dimension (CD)

The complexity of a system can be measured using CD. Grassberger-Procaccia proposed an algorithm to calculate CD [33]. It can be calculated as the slope of the linear scaling region of log⁡*C*(*r*)/log⁡*r*. Here, *C*(*r*) gives the probability that two points chosen randomly are distant *r* or less [34]. In (3), *θ* stands for Heaviside function which gives a “yes or no decision” whether two points *v*_*i*_ and *v*_*j*_ are distant *r* or less [29].

Correlation sum *C*(*r*) is

(3) $$\begin{array}{c} C\left(\;r\;\right)=\frac{1}{N^{2}}\overset{N}{\underset{i=1}{\sum }}\;\overset{N}{\underset{j=1,i\neq j}{\sum }}\theta \left(\;r-\left|\;v_{i}-v_{j}\;\right|\;\right). \end{array}$$

Correlation dimension CD is calculated as

(4) $$\begin{array}{c} CD=\underset{r\to 0}{\lim}\frac{\log C\left(r\right)}{\log\left(r\right)}. \end{array}$$

 
*N* is number of data points in phase space.
 
*r* is radial distance around each reference point *Xi*.
 
*v*_*i*_, *v*_*j*_ are points of the trajectory in the phase space.
 
*θ* is Heaviside function.

#### 2.2.4. Largest Lyapunov Exponent (LLE)

Lyapunov exponent *λ* measures the rate at which the trajectories separate from one another. It gives some dynamic information about the attractor. Largest Lyapunov exponent calculates the chaoticity of a system. LLE (*λ*_max_) of the attractor gives the average rate of convergence or divergence of nearby trajectories in phase space. The commonly used algorithms for calculating LLE were proposed by Wolf et al. [35] and Rosenstein et al. [36]. They are used commonly to extract LLE from EEG data. The mean divergence between neighboring trajectories can be expressed as

(5) $$\begin{array}{c} D\left(\;t\;\right)=De^{\left(\lambda t\right)}. \end{array}$$

*D* is the initial separation between neighboring points and *d*(*t*) represents distance between them in time *t*.

LLE is the slope of average logarithmic divergence of neighboring trajectories.

(6) $$\begin{array}{c} y\left(\;i\;\right)=\frac{1}{\Delta t}\langle \;\ln\left[\;d_{j}\left(\;i\;\right)\;\right]\;\rangle . \end{array}$$

Positive Lyapunov exponent is a good indicator that the system under consideration is chaotic in nature. Here in our analysis, *m* is taken as 10 and *λ* is taken as 1 for calculating the chaotic parameters [3, 37].

### 2.3. Analysis of Data

**Figure 1:**
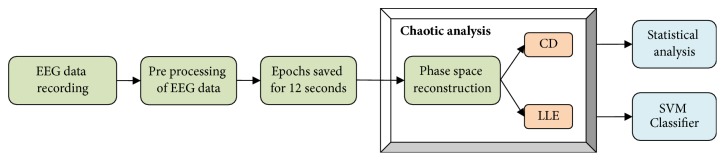
Block diagram of chaotic analysis of EEG samples.

The data obtained by the aforesaid procedures were subjected to statistical analysis. Mann–Whitney *U* test was performed and ROC curves were plotted. Statistical analysis was done in SPSS software. Support Vector Machine (SVM) Classifier was implemented in MATLAB and performance parameters were analyzed to classify the two groups. Sensitivity, specificity, and accuracy were calculated. See Figure 1 for an overview of the whole methodology of this study as a flow diagram.

## 3. Results and Discussion

### 3.1. Statistical Analysis

The CD and LLE of the samples were analyzed. The test of normality by both Kolmogorov-Smirnov (K-S test) and Shapiro-Wilk test revealed that the data was not normally distributed (*p* < 0.05). Hence, nonparametric test was preferable for the comparison of CD and LLE between normal and encephalopathy group.

**Figure 2:**
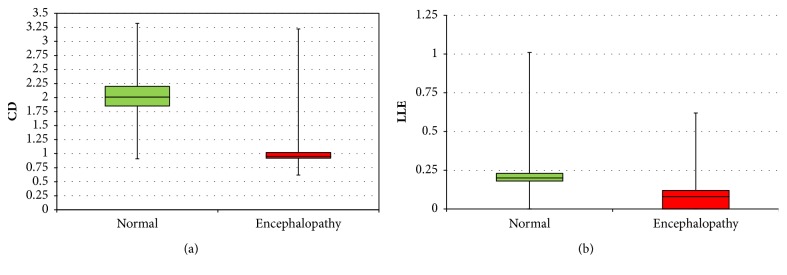
Box plot representing distribution of (a) correlation dimension (CD) and (b) largest Lyapunov exponent (LLE) in EEG of encephalopathic patients and EEG of normal subjects.

**Figure 3:**
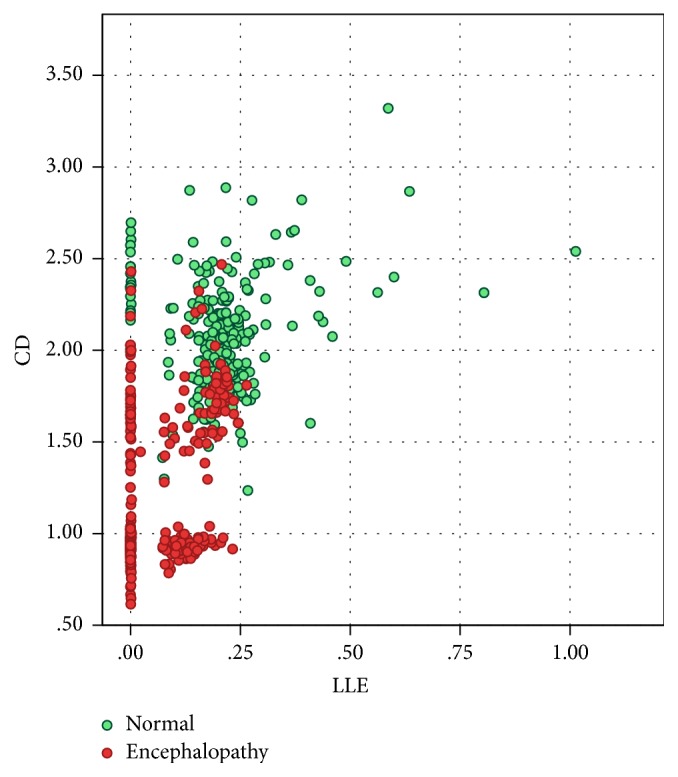
Scatter plot representing distribution of CD and LLE in EEG of encephalopathic patients and EEG of normal subjects.

The median value of CD and LLE for the encephalopathy study group was found to be much less when compared to that of the normal group (refer to Table 3, Figures 2 and 3). Statistical analysis (Mann–Whitney *U* test) was done to test the significance of this result and it proved that the decrease of CD and LLE for the encephalopathy group was statistically significant (CD, *p* value < 0.001; *z*-value = 18.36, and for LLE, *p* value < 0.001; *z*-value = 17.03) (see Table 3). Since *p* values are less than 0.001, it can be inferred that there is a highly significant difference in the CD and LLE values in the normal and encephalopathy groups that cannot be attributed to chance. There is less than 0.1% chance for these groups to have similar chaotic features.

Figure 2 shows distribution of CD and LLE in groups of normal and encephalopathy. Both CD and LLE decrease considerably in metabolic encephalopathy. It shows that both complexity and chaoticity of brain dynamics get lowered due to encephalopathy.

**Figure 4:**
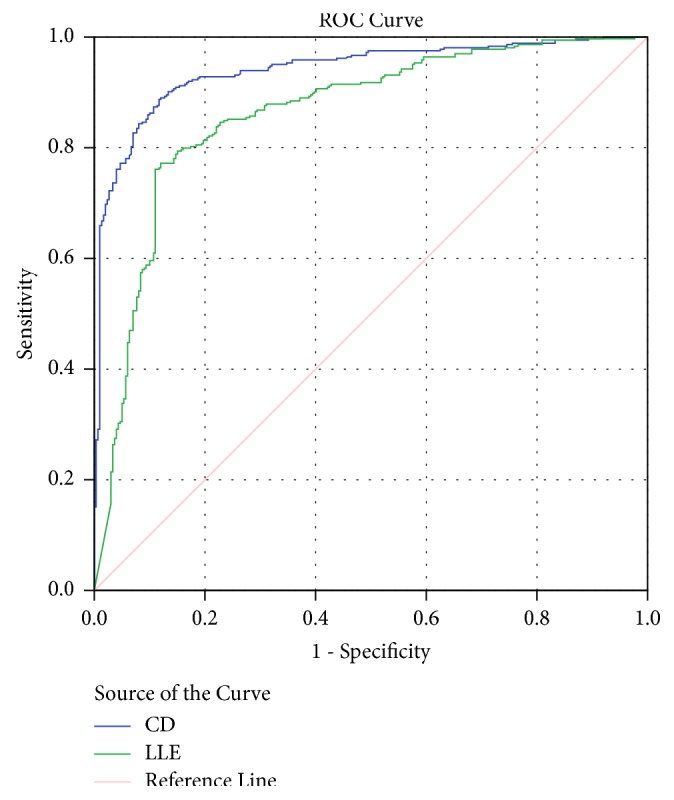
ROC curve in EEG of patients with encephalopathy and EEG of normal subjects.

A scatter diagram was plotted to visualize the pattern of CD and LLE of encephalopathy and normal cases (see Figure 3). The scatter plot gives the impression that the CD and the LLE values tend to be clustered around lower values in the encephalopathic state, whereas they tend to be higher in normal individuals. An ROC curve (Receiver and Operating Characteristic curve) was plotted to derive approximate cut-off values of CD and LLE so as to make a prediction of whether the EEG is normal or one with encephalopathy. The closer the curve follows the left-hand border and the top border of the ROC space, the more accurate the test will be. Here, ROC curve suggests that sensitivity and specificity are both very high for both CD and LLE for normal EEG and EEG of patients with encephalopathy (see Figure 4). The area under curve, AUC, for the plot of CD was 93.2% and for LLE was 85.6%. This suggests that CD may be a better parameter to predict the state of encephalopathy than LLE.

An attempt to deduce cut-off values of CD and LLE for predicting encephalopathy was made. A cut-off of CD value of <1.67 had a sensitivity of 80% and specificity of 93% to predict encephalopathy. A cut-off of <1.91 had a sensitivity of 95% but specificity drops to 64%. For LLE a cut-off value of <0.16 had 80% sensitivity and 80% specificity to predict encephalopathy. A cut-off of <0.197 had a 90% sensitivity and 58% specificity in diagnosing encephalopathy. A specificity of 95% was found if we set a cut-off value of <0.00005 but the sensitivity would be only 30%.

### 3.2. Classifier Performance

Values of 300 EEG epochs of normal and 300 epochs of encephalopathic cases were included for classification. Out of the chaotic features of 600 epochs, 300 were utilized for training and 300 were used for testing the classifier. Detail of data set is given in Table 4. Support vector machine (SVM) classifier was employed for the classification of two groups, giving high accuracy of 97.67%. Confusion matrix for this classification is given in Table 5. It gave 100% specificity and 95.33% sensitivity.

### 3.3. Discussion

We found that CD and LLE decrease considerably in metabolic encephalopathy compared to normal subjects. It shows that both complexity and chaoticity of brain dynamics reduce in encephalopathy. If we consider the brain as an interacting structure of multiple systems and subsystems, during encephalopathy, there is relatively more synchronization between the interacting elements. The higher the level of synchronization between various sources in a network, the lower the complexity.

**Figure 5:**
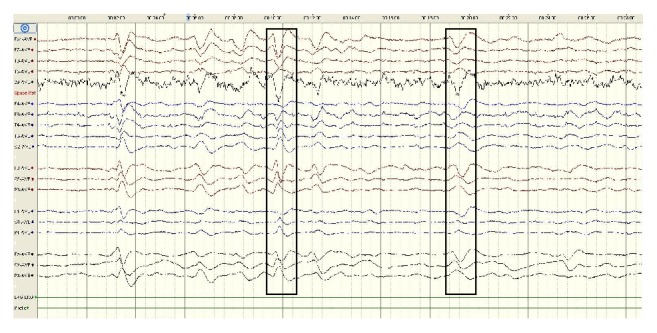
Triphasic wave patterns seen in EEG of encephalopathic patients.

An EEG feature that is consistently seen in encephalopathy is called triphasic wave (TW). Triphasic waves have morphology of a positive deflection preceded and succeeded by a negative deflection. It classically has amplitude more than 70 *μ*V. The TWs are usually seen at nearly 1-2 Hz frequency.  Figure 5 shows the pattern of triphasic waves seen in EEG of encephalopathy in our study sample, though not present in all cases of encephalopathy. These are seen as sharp spikes and it is seen diffusely on all electrodes of both sides but with more prominence in frontal leads [38, 39]. We hypothesise that the synchronous waveforms in these TWs may contribute to the decreased chaoticity and complexity seen in encephalopathic patients, in our analysis. The higher synchrony and hence lower chaoticity exhibited in TWs seen in encephalopathy are analogous to the synchrony exhibited during the hypersynchronous neuronal firing in a seizure [2].

Complexity, the core parameter that we assess using CD and LLE, is eponymous to the measure of randomness in a system of interacting elements. During normal wakeful state of healthy humans, there is a high dimensional complexity, with its interacting systems and subsystems having a weak level of synchronization. In the setting of the condition of encephalopathy, the disease state that we investigated, we can assume that the level of synchronization between elements becomes stronger as evidenced by the low dimensional chaos demonstrated in our results. This finding may be useful to understand better the neurobiologic phenomena in the brain of subjects with encephalopathy. The interactions between the various neurons are less independent in case of encephalopathy compared to healthy brain dynamics, resulting in lesser complexity. CD and LLE give an exact measure of complexity and chaoticity, which may be utilized by neurologists to identify and diagnose the disease condition. These features may also be considered as potential parameters for automated diagnosis of encephalopathy.

However, one limitation of the study should be recognised. The EEG epochs collected from encephalopathy patients were pooled together to compare with the EEG epochs of normal subjects. The different types of encephalopathies or their grades of severity were not analyzed.

## 4. Conclusion

We investigated the chaotic features for EEG in patients with metabolic encephalopathy and found that significant decreased values were observed in both chaotic features, CD and LLE, compared to normal EEG. Results imply that, during the condition of encephalopathy, the complexity and chaoticity, that is, the unpredictability and randomness of the brain EEG, reduce considerably when compared to normal controls. One of the reasons for this phenomenon can be the presence of synchronous triphasic waves seen in encephalopathy. To the best of our knowledge, this is one of the first studies to explore chaotic parameters of EEG and we infer that these features are sensitive to functional alterations of the brain caused by encephalopathy. This study can be extended to utilize chaotic features for detecting various types of encephalopathy and thus in automated diagnosis of the condition.

## Figures and Tables

**Table 1:** Demographic data of normal and encephalopathy group participated in the study.

Group	No. of participants	No. of epochs	Age (Mean; SD)	Gender (M/F)
Encephalopathy	30	331	(57.88; 11.2)	17/13
Normal	30	314	(50.13; 11.3)	16/14

**Table 2:** Various types of encephalopathy included in the database epochs for representing encephalopathy group.

Type of encephalopathy	No. of patients	No. of epochs
Hepatic	17	188
Uremic	13	143

**Table 3:** Median and interquartile range values for CD and LLE of EEGs of normal and encephalopathy group.

Chaotic feature	Group	Sample size	Median	Inter quartile range	Mann-Whitney *U* test	
					*z*	*p*
CD	Normal	314	2.01	1.85–2.20	18.36	<0.001
	Encephalopathy	331	0.95	0.92–1.02		

LLE	Normal	314	0.20	0.18–0.23	17.025	<0.001
	Encephalopathy	331	0.08	0.00–0.12		

**Table 4:** Dataset for training and testing.

	Encephalopathy	Normal	Total
Training	150	150	300
Testing	150	150	300

**Table 5:** Confusion matrix for encephalopathy classification based on chaotic features of EEG.

	Predicted: NO	Predicted: YES	
Actual: NO	143	7	300
Actual: YES	0	150	300

	300	300	

## Data Availability

As per the ethical committee guidelines of the institution, where the study was conducted, it is not permitted to share the EEG data and patients' confidential information in a public repository.

## Supplementary Materials

Supplementary Materials See Figures S1–S3 in the Supplementary Materials for sample EEG epoch recordings of encephalopathic case with triphasic wave and encephalopathic case without triphasics, characterized by nonspecific slowing and a normal EEG, respectively.
