# Supplementary material for: Can Chaotic Analysis of Electroencephalogram Aid the Diagnosis of Encephalopathy?
Source: Neurol Res Int. 2018 May 29;2018:8192820. doi: 10.1155/2018/8192820 (PMC5996471; doi:10.1155/2018/8192820)
Supplement: Supplementary Materials — See Figures S1–S3 in the Supplementary Materials for sample EEG epoch recordings of encephalopathic case with triphasic wave and encephalopathic case without triphasics, characterized by nonspecific slowing and a normal EEG, respectively. [file 8192820.f1.pdf]

SUPPLEMENTARY FILES

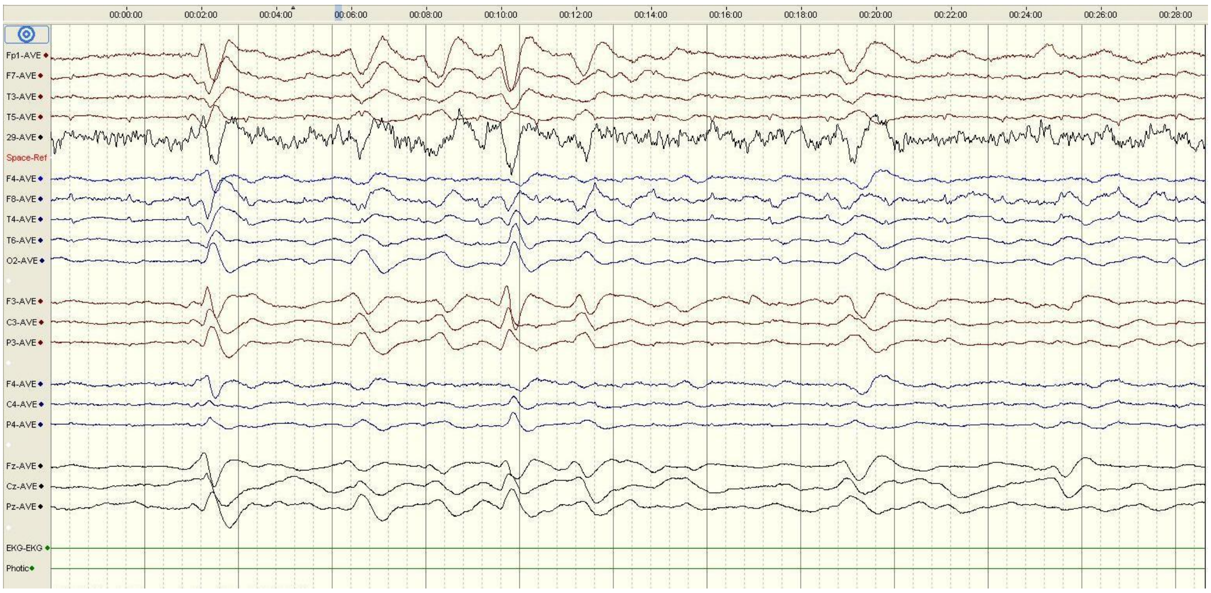

Figure S1: EEG epoch of encephalopathic patient with triphasic waves

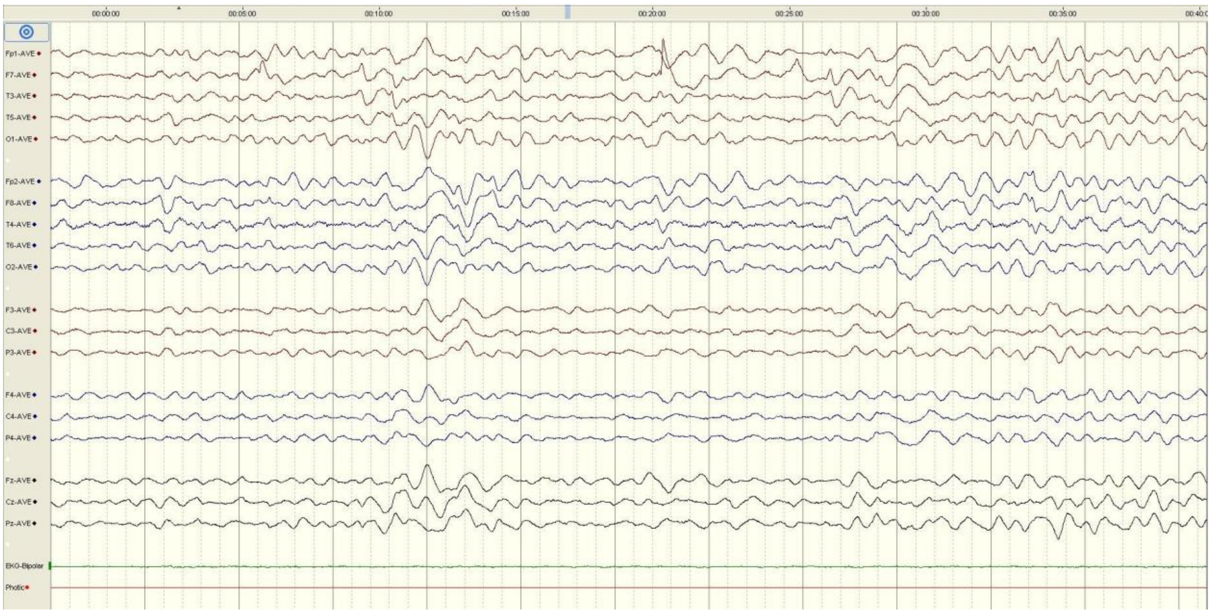

Figure S2: EEG sample showing EEG of encephalopathic patients with marked slowing of EEG

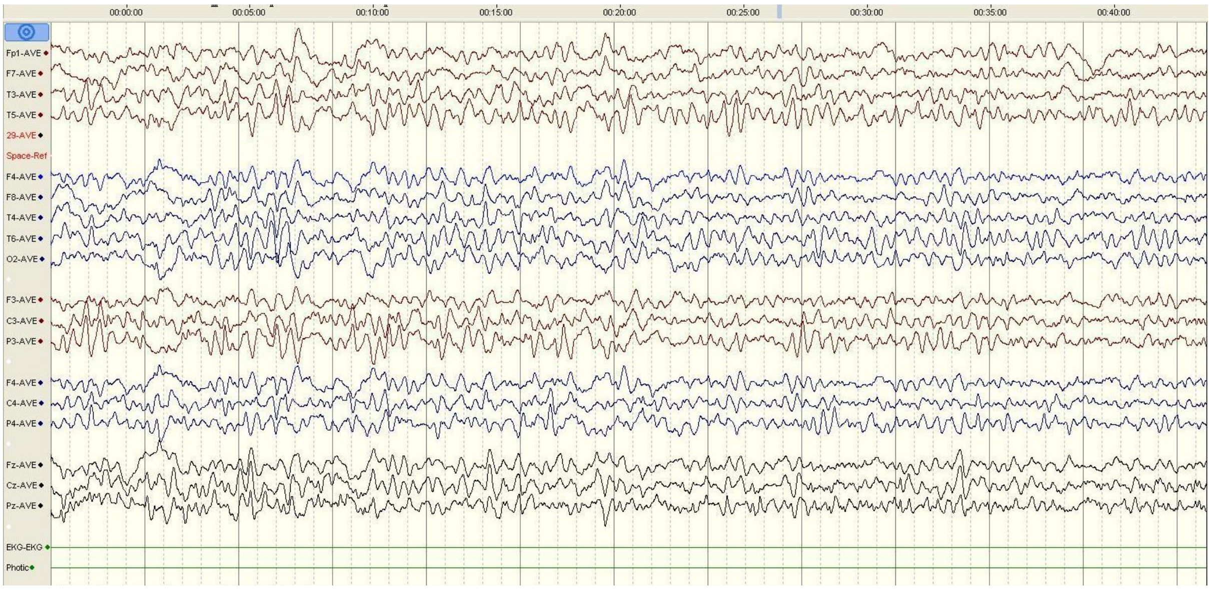

Figure S3: Normal EEG
